# Associations between Two Polymorphisms (FokI and BsmI) of Vitamin D Receptor Gene and Type 1 Diabetes Mellitus in Asian Population: A Meta-Analysis

**DOI:** 10.1371/journal.pone.0089325

**Published:** 2014-03-06

**Authors:** Guofeng Wang, Qingqing Zhang, Ning Xu, Kuanfeng Xu, Jian Wang, Wei He, Tao Yang

**Affiliations:** 1 Department of Endocrinology Medicine, Lianyungang First People’s Hospital, Affiliated Hospital of Xuzhou Medical College, Lianyungang, China; 2 Department of Endocrinology Medicine, The Fist Affiliated Hospital of Nanjing Medical University, Nanjing, China; The University of Hong Kong, Hong Kong

## Abstract

**Background:**

Vitamin D receptor (VDR) gene polymorphisms are possibly involved in the development of type 1 diabetes mellitus (T1DM). However, the results to date have been inconclusive. We performed a meta-analysis to examine the association between 2 polymorphisms (FokI and BsmI) of the VDR gene and T1DM in the Asian population.

**Methods:**

Literature was retrieved from PubMed, Web of Science, CBM, Embase and Chinese databases. Pooled odds ratios (ORs) with 95% confidence intervals (CIs) were calculated using a random or fixed effect model.

**Results:**

In total, 20 papers (BsmI: 13 studies; FokI: 13 studies) were included. In contrast to the FokI polymorphism, the BsmI polymorphism was associated with an increased risk of T1DM in the Asian population (OR = 1.47, 95% CI = 1.13–1.91, P = 0.004 for B vs. b). Upon stratification by regional geography, an increased risk of T1DM in association with the BsmI polymorphism was observed in the East Asian population (OR = 1.97, 95% CI = 1.38–2.83, P<0.001 for B vs. b), whereas the FokI polymorphism was associated with an increased risk of T1DM in the West Asian population (OR = 1.45, 95% CI = 1.12–1.88, P = 0.004 for F vs. f).

**Conclusions:**

Our meta-analysis suggests that the BsmI polymorphism may be a risk factor for susceptibility to T1DM in the East Asian population, and the FokI polymorphism is associated with an increased risk of T1DM in the West Asian population. However, because the study size was limited, further studies are essential to confirm our results.

## Introduction

Type 1 diabetes mellitus (T1DM) is a T-cell-mediated autoimmune disease, resulting from autoimmune destruction of β-cells of the pancreas. Although the complicated mechanisms involved in T1DM remain unclear, it is well known that T1DM is caused by complex interactions between many genetic and environmental factors. So far, several gene polymorphisms have been demonstrated to be associated with the risks of T1DM, including the human lymphocyte antigen (HLA) gene, the regulatory region of the insulin gene and the interleukin-1 receptor type 1 (IL1R1) gene, cytotoxic T lymphocyte associated-4 (CTLA-4) gene and the vitamin D receptor (VDR) gene. Among these susceptibility genes, the VDR gene is one of the most widely studied and increasing evidence suggests that vitamin D and its receptor are possibly related to T-cell-mediated autoimmune disease and influence susceptibility to T1DM.

The VDR is found in almost all cells of the immune system, especially the antigen-presenting cells (macrophages and dendritic cells) and activated T lymphocytes. It is increasing acknowledged that vitamin D is a potent modulator of the immune system and is involved in regulating cell proliferation and differentiation, lymphocyte activation and cytokine production [1], [2], and it plays a central role in the pathogenesis and progression of T1DM. Previous epidemiologic and experimental data indicate that taking vitamin D supplements in early childhood and high vitamin D intake may be inversely associated with the risk of T1DM incidence, and treatment with large doses of vitamin D over long periods in non-obese diabetic NOD mice has been known to prevent the disease [3]. Because vitamin D exerts it action at a cellular level through binding to the VDR, which is an intracellular hormone receptor belonging to the steroid hormone receptor superfamily, the VDR gene has become a candidate gene for T1DM.

The human VDR gene is located on chromosome 12q12–q14. It has at least 5 promoter regions, 8 protein-coding exons and 6 untranslated exons, which are alternatively spliced into FokI (in exon 2), BsmI and ApaI (both in intron 8) and TaqI (in exon 9). In the past decades, a series of studies have investigated the association between FokI (rs10735810) and BsmI (rs1544410) common single nucleotide polymorphisms (SNPs) and T1DM risk but the findings have been conflicting. In 2006, Guo et al [4] did not identify any genetic variant associated with T1DM in either case-control studies or family transmission. But subsequent reanalysis of this data by Ponsonby et al [5] in 2008, incorporating adjustment for regional ultraviolet(UV) radiation, supported an association between VDR gene polymorphisms and T1DM risk. In 2012, Zhang et al [6] further explored the association between VDR gene polymorphisms and T1DM by ethnical subgroup and confirmed that the BsmI polymorphism was associated with an increased risk of T1DM in the Asian subgroup, with no significant association found in other populations for FokI polymorphisms. However these results were still inconclusive and did not report the regional geography-specific effects on the risk of T1D, In addition, data from a number of Asian studies published in the past few years [7]–[10], should also be included.

To confirm the association between 2 common polymorphisms (FokI and BsmI) in the VDR gene and T1DM susceptibility in Asian population, and to investigate regional geography-specific effects with T1DM, we performed an updated meta-analysis.

## Materials and Methods

### Literature and Search Strategy

To identify studies that investigated the association between T1DM susceptibility and VDR polymorphisms, the databases PubMed, Web of Science, CBM, Embase, Chinese Biomedical Literature Database, Chinese National Knowledge Infrastructure (CNKI) and Chinese Wanfang Data were searched independently by 2 reviewers. The last updated search was performed on March 29, 2013. The following search terms were used: (vitamin D receptor or VDR) AND (polymorphism or variation or mutation) AND (type 1 diabetes mellitus or IDDM or autoimmune diabetes). Studies published in English and Chinese were considered in our meta-analysis.

### Inclusion Criteria and Data Extraction

The studies were included only if they met all the following inclusion criteria: (1) evaluation of 2 polymorphisms in the VDR gene and T1DM risk; (2) a case-control study design and (3) genotype distributions in both cases and controls available for estimating an odds ratio (OR) with 95% confidence interval (CI). Studies were excluded if they were case-only reports or review papers or the study was based on pedigree data. When the data were duplicated and had been published more than once, only the most recent and complete study was used.

The following information was extracted from each study: (1) name of first author; (2) year of publication; (3) country of origin; (4) ethnicity of the study population; (5) gender distribution and mean age of subjects in cases and controls; (6) mean age of onset in cases; (7) genotype distributions in cases and controls; (8) P values for the test of Hardy–Weinberg equilibrium (HWE) in controls; (9) diagnostic criteria and (10) manner in which the controls were selected. Two authors independently assessed the articles for compliance with the inclusion criteria, and disagreement was followed by discussion or a third reviewer was asked to assess those articles until consensus was reached. When further information was required from a potentially relevant manuscript, the corresponding authors were contacted by the reviewers.

### Quality Assessment of Included Studies

The quality of the included studies was also independently assessed by 2 authors using the procedure known as ‘extended quality score’, which was used in the study by Xu et al [11] and based on the recommendations of the MOOSE guidelines and other related meta-analytic studies. The procedure with 11 items stems from epidemiological and genetic considerations and the full score is 14 points. Studies were categorized as ‘high’ quality if the score was ≥11 points, ‘medium’ if the score was <11 and ≥7 points and ‘poor’ if the score was <7 points.

### Statistical Analysis

The strength of association between VDR polymorphisms and T1DM risk was assessed by ORs with a 95% CI. The HWE of the genotype distribution of controls was assessed via the χ2 goodness-of-fit test. The genetic models evaluated for pooled ORs of these polymorphisms were the additive model (A vs. G), the codominant model (AA vs. AG and AA vs. GG), the dominant model (GG vs. AA+AG) and the recessive model (AA vs. AG+GG). Statistical analysis was conducted by subgroup divisions on the basis of regional geography. The significance of the pooled ORs was determined using Z tests (with P<0.05 considered statistically significant). The heterogeneity among studies was evaluated by the Q statistic test and I^2^ statistic test. P values <0.05 or I^2^>50% indicated that heterogeneity existed among studies, and the pooled OR of each study was calculated by the random effects model (REM), because it is more appropriate when there is significant heterogeneity. Otherwise, the fixed effects model FEM was used. If heterogeneity existed among studies, we used meta-regression to explore the source. The potential publication bias was estimated using Begg’s funnel plot and Egger’s test (with P<0.05 considered statistically significant). If publication bias existed, the Duval and Tweedie nonparametric ‘trim and fill’ method was used to adjust the findings. Sensitivity analysis was performed to evaluate quality and consistency of the results. Data analysis was performed using STATA version 11.0 software.

## Results

### Eligible Studies and their Characteristics

**Figure 1:**
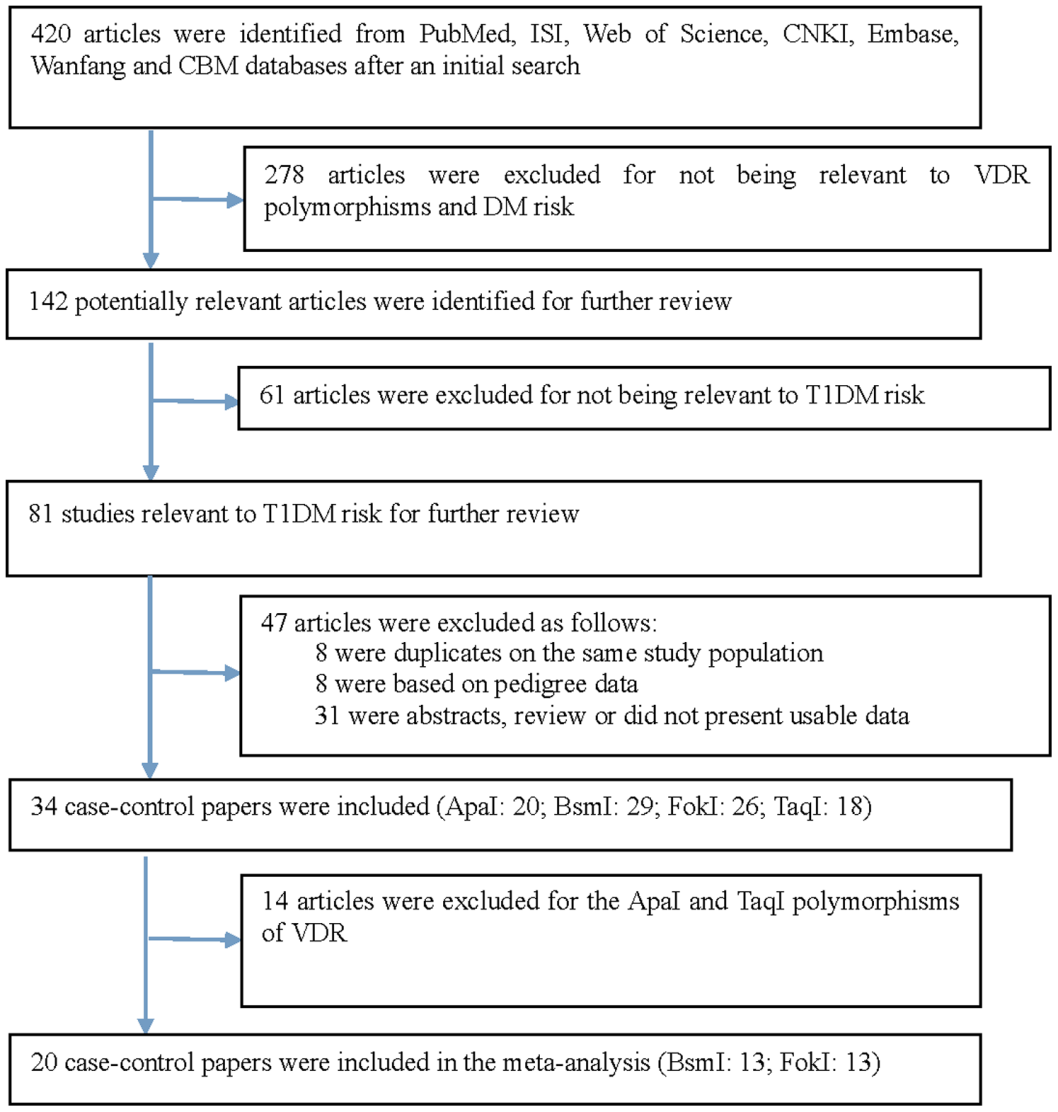
Selection of articles for inclusion in the meta-analysis.

The detailed steps of our literature search are shown in Figure 1. Based on the search strategy and inclusion criteria, 20 papers were included in the meta-analysis of the association between polymorphisms in the VDR gene and T1DM risk between 1996 and March 29, 2013[7]–[10], [12]–[27]. There were 13 studies (1,973 cases and 1,986 controls) for the BsmI polymorphism and 13 studies (2,538 cases and 2.679 controls) for the FokI polymorphism.

The characteristics of the studies are listed in Table S1. Genotype and allele distributions for each study are listed in Table 1. Among these studies, T1DM was diagnosed according to different criteria. World Health Organization criteria were used in 10 studies; American Diabetes Association criteria were used in 4 studies; National Diabetes Data Group & International Work Group 1997 criteria were used in 1 study and 2 studies did not note the diagnostic criteria. All the included studies contained analysis of the VDR gene polymorphism by polymerase chain reaction–restriction fragment length polymorphism (PCR–RFLP). At the same time, all studies categorized the score as ‘medium’ or ‘high’ quality, with the exception of 2 studies. [14], [23], in which the detailed quality assessment criterion and quality score are listed in Tables S2 and S3.

**Table 1:** Distribution of VDR genotype and the allele among T1DM patients and controls.

First author	Country	Year	Case					Control					HWE
BsmI			BB	Bb	bb	B	b	BB	Bb	bb	B	b	
Chang [12]	China	2000	4	16	137	24	290	1	16	231	18	478	Yes
Motohashi [15]	Japan	2003	12	64	127	88	318	1	49	172	51	393	Yes
Liu [21]	China	2003	31	103	91	165	285	110	305	403	525	1111	No
Shen [22]	China	2004	0	11	24	11	59	0	7	45	7	97	Yes
Xiao [26]	China	2006	1	4	49	6	102	0	1	81	1	163	No
Shi [24]	China	2007	1	20	22	22	64	1	18	177	20	372	Yes
Shimada [25]	Japan	2008	32	165	577	229	1319	7	121	471	135	1063	Yes
Israni [18]	India	2009	79	120	37	278	194	56	94	47	206	188	Yes
Kocabas [19]	Turkey	2010	1	14	75	16	164	0	16	70	16	156	Yes
Cheng [27]	China	2010	43	11	0	97	11	82	19	1	183	21	Yes
Gogas Yavuz [17]	Turkey	2011	20	57	40	97	137	14	59	61	87	181	Yes
Mohammadnejad [8]	Iran	2012	11	36	40	58	116	9	45	46	63	137	Yes
Bonakdaran [7]	Iran	2012	14	26	29	54	84	16	11	40	43	91	Yes
FokI			FF	Ff	ff	F	f	FF	Ff	ff	F	f	HWE
Ban [13]	Japan	2001	52	52	6	156	64	82	138	30	302	198	No
Yokota [14]	Japan	2002	50	46	12	146	70	41	59	20	141	99	Yes
Shen [22]	China	2004	5	14	16	24	46	18	24	10	60	44	Yes
Liao [20]	China	2005	18	49	18	85	85	64	74	28	202	130	Yes
Du [16]	China	2008	64	130	47	258	224	123	189	68	435	325	Yes
Sheng [23]	China	2009	39	36	5	114	46	25	41	14	91	69	Yes
Israni [18]	India	2009	142	79	15	363	109	116	76	5	308	86	Yes
Kocabas [19]	Turkey	2010	32	57	1	121	59	18	52	16	88	88	Yes
Xie [9]	China	2012	125	211	101	461	413	135	209	86	479	388	Yes
Sahin [10]	Iran	2012	54	31	0	139	31	43	28	9	114	46	No
Gogas Yavuz [17]	Turkey	2011	61	46	10	168	66	60	63	11	183	85	Yes
Mohammadnejad [8]	Iran	2012	49	33	5	131	43	55	40	5	150	50	Yes
Bonakdaran [7]	Iran	2012	38	25	6	101	37	18	20	7	56	34	Yes

### Quantitative Synthesis

A summary of the meta-analysis of the association between VDR gene polymorphisms and T1DM risk is provided in Table 2.

**Table 2:** Tabel 2. Summary ORs and 95% CIs of the association between VDR gene polymorphism and type 1 diabetes mellitus risk.

SNPs	Regional geograph	A vs a		AA vs Aa+aa		aa vs AA+Aa		AA vs Aa		AA vs aa	
		OR(95%CI)	P	OR(95%CI)	P	OR(95%CI)	P	OR(95%CI)	P	OR(95%CI)	P
BsmI	overall	1.47(1.13,1.91)	0.004	1.72(1.16,2.57)	0.004	0.44(0.29,0.65)	<0.001	1.45(1.09,1.91)	0.01	2.23(1.62,3.07)	<0.001
	East Asian	1.97(1.38,2.83)	<0.001	3.20(1.39,7.33)	<0.001	0.36(0.20,0.63)	<0.001	1.72(0.91,3.26)	0.022	4.71(2.44,9.08)	0.038
	West Asian	0.90(0.52,1.56)	0.704	1.32(0.83,2.10)	0.704	0.52(0.20,1.36)	0.185	1.75(1.09,2.82)	0.097	1.75(1.07,2.85)	0.027
FokI	overall	1.14(0.92,1.41)	0.234	1.16(0.90,1.51)	0.26	0.77(0.49,1.22)	0.271	1.16(0.91,1.48)	0.226	1.11(0.91,1.36)	0.789
	East Asian	1.00(0.74,1.35)	0.995	0.99(0.65,1.51)	0.961	0.99(0.65,1.50)	0.947	0.90(0.69,1.43)	0.967	0.96(0.73,1.26)	0.657
	West Asian	1.45(1.12,1.88)	0.004	1.46(1.11,1.92)	0.006	0.24(0.06,1.02)	0.052	1.44(1.06,1.96)	0.019	1.45(1.12,1.88)	0.004

SNPs: single nucleotide polymorphisms.

A represent B allele and a represent b allele for BsmI, A present F allele and a represent f allele for FokI, respectively.

### BsmI Polymorphism

**Figure 2:**
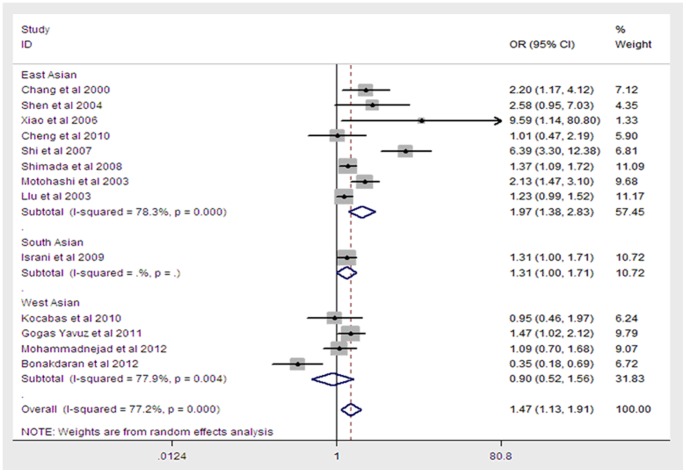
Meta-analysis for the association between T1DM risk and the VDR BsmI polymorphism (B vs b). Each study was shown by a point estimate of the effect size (OR) (size inversely proportional to its variance) and its 95% confidence interval (95%CI) (horizontal lines). The white diamond denotes the pooled OR.

A total of 1,973 T1DM cases and 1,986 controls in 13 case-control studies were included in the meta-analysis for the relationship between the BsmI polymorphism and T1DM risk. Of these, 8 case-control studies were from the East Asian population, 1 was from the South Asian population and 4 were from the West Asian population. Our meta-analysis showed a significant overall association between the BsmI polymorphism and T1DM risk in all models (additive REM: OR = 1.47, 95% CI = 1.13–1.91, P = 0.004(Figure 2); dominant REM: OR = 0.44, 95% CI = 0.29–0.65, P<0.001; recessive FEM: OR = 1.72, 95% CI = 1.16–2.57, P = 0.004; codominant FEM: OR = 1.45, 95% CI = 1.09–1.91, P = 0.01 for BB vs. Bb; OR = 2.23, 95% CI = 1.62–3.07, P<0.001 for BB vs. bb). Upon stratification by regional geography, significantly increased risks were found among the East Asian population (additive REM: OR = 1.97, 95% CI = 1.38–2.83, P<0.001; dominant REM: OR = 0.36, 95% CI = 0.20–0.63, P<0.001; recessive FEM: OR = 3.20, 95% CI = 1.39–7.33, P≤0.001;codominant FEM: OR = 1.75, 95% CI = 1.09–2.82, P = 0.022 for BB vs. Bb; OR = 4.71, 95% CI = 2.44–9.08, P<0.001 for BB vs. bb). However, there were no statistically significant correlations between BsmI polymorphisms and the South or West Asian populations. Thus, the East Asian variant allele B carriers may have a higher risk of T1DM. A summary of the results of the genetic model comparisons is shown in Table 2.

### FokI Polymorphism

**Figure 3:**
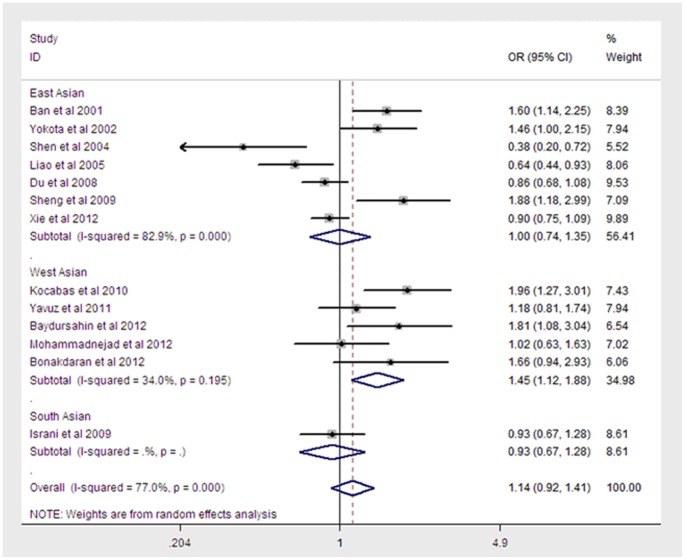
Meta-analysis for the association between T1DM risk and the VDR FokI polymorphism(F vs f). Each study was shown by a point estimate of the effect size (OR) (size inversely proportional to its variance) and its 95% confidence interval (95%CI) (horizontal lines). The white diamond denotes the pooled OR.

A total of 2,538 cases and 2,679 controls were included in the meta-analysis for the relationship between the FokI polymorphism and T1DM risk. Of these, 5 studies were from the West Asian population, 7 were from the East Asian population and 1 was from the South Asian population. Overall, the FokI polymorphism was not associated with the risk of T1DM (additive REM: OR = 1.14, 95% CI = 0.92–1.41, P = 0.234 (Figures 3); dominant REM: OR = 0.77, 95% CI = 0.49–1.22, P = 0.271; recessive REM: OR = 1.16, 95% CI = 0.90–1.51, P = 0.26; codominant REM: OR = 1.16, 95% CI = 0.91–1.48, P = 0.226 for FF vs. Ff; OR = 1.11, 95% CI = 0.91–1.36, P = 0.31 for FF vs. ff). However, upon stratification by regional geography, the FokI polymorphism was associated with the risk of T1DM in the West Asian population (additive REM: OR = 1.45, 95% CI = 1.12–1.88, P = 0.004; dominant REM:OR = 0.24, 95% CI = 0.06–1.02, P = 0.0271; recessive REM: OR = 1.46, 95% CI = 1.11–1.92, P = 0.006;codominant REM: OR = 1.44, 95% CI = 1.06–1.96, P = 0.019 for FF vs. Ff; OR = 1.45, 95% CI = 1.12–1.88, P = 0.004 for FF vs. ff). A summary of the genetic model comparisons is shown in Table 2.

### Sensitivity Analysis and Publication Bias

Sensitivity analysis was performed to assess the influence of each individual study on the pooled OR by repeating the meta-analysis sequentially excluding 1 study at a time; the analysis results suggested that the pooled ORs were not significantly affected (data not shown), indicating a statistically robust result. The funnel plots were inspected for geometry and were found to be symmetrical, and no publication bias was detected by either Begg’s funnel plot (Figure S1) or the Egger’s test in each meta-analysis.

### Heterogeneity Analyses

Significant heterogeneity was observed in most genetic model analyses (Table S4). We observed the pattern of heterogeneity: for BsmI, 9 out of 13 studies showed a positive result, and for FokI, 8 out of 13 studies showed a positive result. Thus, the heterogeneity in this meta-analysis may have resulted from the inclusion of a mixture of studies showing positive, null and inverse associations. To investigate this further, we used meta-regression analysis to determine the source of heterogeneity in publication year, ratio of males in cases and controls, mean age or onset age in cases, sample size in cases and controls, diagnostic criterion and quality scoring. Univariate meta-regression analysis showed that none of the tested covariates could explain the observed heterogeneity. Moreover, when the data were stratified by regional geography, the heterogeneity was not significantly decreased or eliminated.

## Discussion

The two polymorphisms (BsmI, FokI) in the VDR gene have been suggested as potential genetic factors for T1DM. However, the results from published studies are still inconsistent. The discrepancies may be partly attributed to differing genetic backgrounds and environment among various populations. To adjust for potential confounding factors in this meta-analysis, we examined the data by regional stratification across Asian populations. Our meta-analysis found that allele B was significantly associated with T1DM in the East Asian population, whereas FokI polymorphisms were associated with an increased T1DM risk in the West Asian population. Therefore, the BsmI polymorphism may increase the risk of T1DM in the East Asian population and the FokI polymorphism may increase the risk of T1DM in the West Asian population. However, considering the heterogeneity and the limited size of included studies, these results should be interpreted cautiously. Undoubtedly, further large scale case-control studies should be performed.

In the present study, the heterogeneity was significant under some genetic models. Moreover, the heterogeneity was not remarkably decreased after stratified analysis or upon exclusion of the studies that deviated from the HWE. These findings were different from those of Zhang et al. [6]. We used the meta-regression analysis to attempt to identify sources of heterogeneity; however, we could not find the source among ethnicity, HWE, sample sizes, quality scoring, diagnosis and publication date. Because of the incomplete data, we could not further explore the source of heterogeneity and the impact of these features on the results even by contacting the corresponding authors. However, we hypothesize that the pattern of heterogeneity in this meta-analysis may have resulted from the inclusion of a mixture of studies showing positive, null and inverse associations instead of all studies showing the same association between VDR gene and T1DM. In addition, the sensitivity analysis proved the robustness of our meta-analysis. Considering that environmental factors influence the circulating levels of active vitamin D forms and may modulate genotype-related risk, gene–environment interaction may be the cause of heterogeneity in this meta-analysis.

Present study has some limitations that require consideration. First, studies have shown that the association between VDR polymorphisms and disease can vary by either past sun exposure or vitamin D level. Our meta-analysis was based on estimates without adjusting for sun exposure or dietary vitamin D intake, which may be one of the potential limitations. Second, lack of further adjustments of environmental risk factors, other covariables, the potential gene environment interactions might bias the present results. Third, because we only searched papers in English or Chinese, the completeness of evidence is impeded by language bias. Fourth, in the stratified analysis by regional geography, only 2 studies concerning the relationship between the BsmI polymorphism and the West Asian population were included; with such a small size, the possibility of finding a reliable association is limited. Thus, the conclusion on the relationship between the BsmI polymorphism and the West Asian population requires further confirmation. Moreover, only 1 study on the South Asia population was currently available for our meta-analysis, and stratified analysis for the South Asian population was not available. Thus, additional studies are needed to evaluate the effect of these functional polymorphisms on T1DM risk in West and South Asian population.

In conclusion, the current meta-analysis indicates that the BsmI polymorphism may contribute to T1DM pathogenesis and may help to explain individual differences in the susceptibility to T1DM in the East Asian population. Furthermore, it shows that the FokI polymorphism may confers susceptibility to T1DM in the West Asian population. However, the power is not enough, and large, well-designed epidemiological studies are necessary to carefully explore the roles of VDR gene polymorphisms in the pathogenesis of T1DM in the Asian population.

## Supporting Information

Figure S1 **Begg’s funnel plot analysis for the comparison of the FokI (A), BsmI (B) alleles.** Each point represents an independent study for the indicated association. p value of Begg’s test was 0.695 and 0.225, respectively (continuity corrected).
(TIF)

Table S1 **Characteristics of the studies included in the meta-analysis.** “NA” means that the data were not available.
(DOC)

Table S2 **Extended quality assessment criteria.** Note: the full score is 14 points, if an original study has a quality score greater than or equal to 11 points, it is high-quality designed; and if a quality score less than 7 points, poor-quality designed; and if a quality score greater than or equal to 7 points and less than 11 points, medium-quality designed.
(DOC)

Table S3 **Quality score of included study.** HWE: Hardy-Weinberg equilibrium.
(DOC)
