# Supplementary figures and images for: Associations between Two Polymorphisms (FokI and BsmI) of Vitamin D Receptor Gene and Type 1 Diabetes Mellitus in Asian Population: A Meta-Analysis
Source: PLoS One. 2014 Mar 6;9(3):e89325. doi: 10.1371/journal.pone.0089325 (PMC3945782; doi:10.1371/journal.pone.0089325)

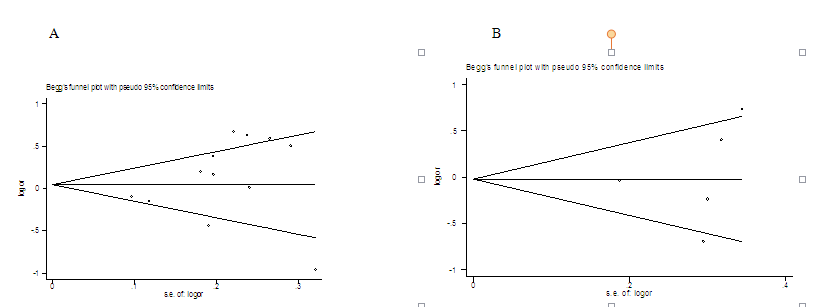

Supplement: Figure S1 — Begg’s funnel plot analysis for the comparison of the FokI (A), BsmI (B) alleles. Each point represents an independent study for the indicated association. p value of Begg’s test was 0.695 and 0.225, respectively (continuity corrected). (TIF) [file pone.0089325.s001.tif]
