# Supplementary material for: Associations between Two Polymorphisms (FokI and BsmI) of Vitamin D Receptor Gene and Type 1 Diabetes Mellitus in Asian Population: A Meta-Analysis
Source: PLoS One. 2014 Mar 6;9(3):e89325. doi: 10.1371/journal.pone.0089325 (PMC3945782; doi:10.1371/journal.pone.0089325)
Supplement: Table S1 — Characteristics of the studies included in the meta-analysis. “NA” means that the data were not available. (DOC) [file pone.0089325.s002.doc]

**Table S1 :Characteristics of the studies included in the meta-analysis.**

| Author | Year | Country | Polymorphism | Case |  |  | Control |  |  | Diagnosis | Source of controls |
| --- | --- | --- | --- | --- | --- | --- | --- | --- | --- | --- | --- |
|  |  |  |  | N | M% | Age(year) | N | M% | Age(year) |  |  |
| Chang | 2000 | China | Bsml | 157 | NR | 8.8±5.6 | 248 | NR | NR | National Diabetes Data Group&International Work Group,1997 | Regional match |
| Ban | 2001 | Japan | FokI | 110 | 45.5 | 26.0±3.7 | 250 | NR | NR | ADA | People without a family history of diabetes/autoimmune disease |
| Yokota | 2002 | Japan | FokI | 108 | 38 | 8.9(0.4-18) | 120 | NR | NR | Unknown | Nonrelated nondiabetic subjects |
| Motohashi | 2003 | Japan | Bsml | 203 | 47.3 | 34.6±16.9 | 222 | NR | 44.4±13.7 | WHO | Unrelated nondiabetic persons |
| Liu | 2003 | China | Bsml | 54 | 52 | 21.36±11. | 82 | 52 | 34.5±8.0 | WHO | Health undergoing check-ups |
| Shen | 2004 | China | Bsml,  FokI | 35 | 48.6 | 29.13±15.2 | 52 | 59.6 | 36.0±4.9 | WHO | Regional match |
| Liao | 2005 | China | FokI | 85 | 57.6 | 26.8±13.9  (1.5-68) | 166 | 62.7 | 61.8±10.8  (37-80) | WHO | Convenience samples |
| Xiao | 2006 | China | Bsml | 54 | 51.9 | 15.5±7.8 | 82 | 52.4 | 34.5±8.0 | WHO | unrelated healthy volunteers |
| Shi | 2007 | China | Bsml | 43 | 65 | 38±10 | 196 | 65.8 | NR | WHO | Healthy controls |
| Shimada | 2008 | Japan | Bsml | 774 | 48 | 29.8(1-78) | 599 | NR | NR | ADA | Health subjects |
| Du | 2008 | China | FokI | 241 | 55 | 24.7±13.2 | 380 | NR | 61.4±9.8 | WHO | regional match |
| Sheng | 2009 | China | FokI | 80 | NR | 29±15 | 80 | NR | 35±5 | Unknown | Health undergoing check-ups |
| Israni | 2009 | India | Bsml  FokI | 236 | 55.5 | 14.74±7.57(F)  16.89±7.25(M) | 194 | 58.9 | 30.1±10.2 | ADA | Health controls |
| Kocabas | 2010 | Turkey | Bsml  FokI | 90 | 53.3 | 11.7±3.82 | 86 | 33.3 | 28.9±5.9 | WHO | Healthy medical students and hospital staff |
| Cheng | 2010 | China | Bsml | 54 | NR | NR | 102 | NR | NR | WHO | Regional match |
| Gogas Yavuz | 2011 | Turkey | Bsml  FokI | 117 | 48.7 | 27.6±7.3 | 134 | NR | NR | ADA | Regional match |
| Mohammadnejad | 2012 | Iran | Bsml  FokI | 87 | 26.4 | 27.93±10.86 | 100 | 50 | 28.58±7.40 | WHO | Health subjects |
