# Supplementary material for: Associations between Two Polymorphisms (FokI and BsmI) of Vitamin D Receptor Gene and Type 1 Diabetes Mellitus in Asian Population: A Meta-Analysis
Source: PLoS One. 2014 Mar 6;9(3):e89325. doi: 10.1371/journal.pone.0089325 (PMC3945782; doi:10.1371/journal.pone.0089325)
Supplement: Table S2 — Extended quality assessment criteria. Note: the full score is 14 points, if an original study has a quality score greater than or equal to 11 points, it is high-quality designed; and if a quality score less than 7 points, poor-quality designed; and if a quality score greater than or equal to 7 points and less than 11 points, medium-quality designed. (DOC) [file pone.0089325.s003.doc]

**Table S2: Extended quality assessment criteria.**

| No. | Items for assessment | Full score | Descriptions/Guidelines for scoring instruments |
| --- | --- | --- | --- |
| Q1 | Study Design | 1 | Give 1 point if being Prospective study; Otherwise give 0 point. |
| Q2 | Diagnostic criteria | 1 | Give 1 point if T1DM was diagnosed based on WHO /ADA/National Diabetes Data & International Work Group, 1997 criteria; Otherwise give 0 point.  . |
| Q3 | Age | 1 | Give 1 point if matching between cases and controls; Give 0 point if "No". |
| Q4 | Gender | 1 | Give 1 point if matching between cases and controls; Give 0 point if "No". |
| Q5 | Region | 1 | Give 1 point if the objects were composed of same ethnical origin/population, or different populations were analysed separately; Give 0 point if different populations were mixed up (such as using ambiguous, country/not ethnical description in the article). |
| Q6 | Ethnicity | 1 | Give 1 point if the objects were consistent and matched ethnically between cases and controls; Give 0 point if "No". |
| Q7 | Hardy-Weinberg equilibrium | 2 | Give 2 points if being in Hardy-Weinberg equilibrium; Otherwise, give 1 point if 0.05 > P > 0.01; give 0 point if P < 0 .01. |
| Q8 | Experimental method | 1 | Give 1 point if the acknowledged or strict experimental method was described; Give 0 point if "No". |
| Q9 | Bias in data processing | 1 | Give 1 point if the raw data were rechecked by other independent investigators or used “blind” during the experimental and statistical periods. |
| Q10 | Source of Control Subjects | 1 | Give 1 points for population-based control subjects; give o point for hospital-based control subjects |
| Q11 | Sample size for patients | 3 | 1. ≥1000: 3 points; 2. 500-999: 2 points; 3. 200-499: 1 Scores; 4. <200: 0 Score. |
| Total score 14 The maximum possible score was 14 points.  . | | | |
