# Supplementary material for: Associations between Two Polymorphisms (FokI and BsmI) of Vitamin D Receptor Gene and Type 1 Diabetes Mellitus in Asian Population: A Meta-Analysis
Source: PLoS One. 2014 Mar 6;9(3):e89325. doi: 10.1371/journal.pone.0089325 (PMC3945782; doi:10.1371/journal.pone.0089325)
Supplement: Table S3 — Quality score of included study. HWE: Hardy-Weinberg equilibrium. (DOC) [file pone.0089325.s004.doc]

**Table S3: Quality score of included study.**

| study | Study design | Diagnostic criteria | Age | Gender | Region | Ethnicity | HWE | Experimental method | Bias in data processing | Source of Control Subjects | Sample size for patients | Total  score |
| --- | --- | --- | --- | --- | --- | --- | --- | --- | --- | --- | --- | --- |
| Chang | 1 | 1 | 0 | 0 | 1 | 1 | 2 | 1 | 0 | 1 | 1 | 9 |
| Liu | 0 | 1 | 1 | 1 | 1 | 1 | 0 | 1 | 1 | 0 | 0 | 7 |
| Shen | 0 | 1 | 1 | 1 | 1 | 1 | 2 | 1 | 0 | 0 | 0 | 8 |
| Xiao | 1 | 1 | 0 | 1 | 1 | 1 | 0 | 1 | 1 | 1 | 0 | 8 |
| Shi | 0 | 1 | 0 | 0 | 1 | 1 | 2 | 1 | 0 | 0 | 0 | 6 |
| Cheng | 0 | 1 | 0 | 1 | 1 | 1 | 2 | 1 | 1 | 1 | 0 | 9 |
| Motohashi | 1 | 1 | 1 | 1 | 1 | 1 | 2 | 1 | 1 | 0 | 1 | 11 |
| Shimada | 1 | 1 | 0 | 0 | 0 | 1 | 2 | 1 | 0 | 1 | 2 | 9 |
| Israni | 0 | 1 | 0 | 1 | 1 | 1 | 0 | 1 | 1 | 1 | 0 | 7 |
| Bonakdaran | 0 | 1 | 0 | 0 | 1 | 1 | 2 | 1 | 1 | 1 | 0 | 8 |
| Mohammadnejad | 1 | 1 | 0 | 0 | 1 | 1 | 2 | 1 | 1 | 1 | 0 | 9 |
| Yavuz | 1 | 1 | 1 | 1 | 0 | 1 | 2 | 1 | 1 | 0 | 0 | 9 |
| Kocabas | 1 | 1 | 1 | 1 | 1 | 1 | 2 | 1 | 0 | 0 | 0 | 9 |
| Ban | 1 | 1 | 1 | 0 | 1 | 1 | 0 | 1 | 0 | 1 | 0 | 7 |
| Yokota | 1 | 0 | 0 | 0 | 1 | 1 | 0 | 1 | 0 | 0 | 0 | 4 |
| Liao | 1 | 1 | 0 | 1 | 1 | 1 | 2 | 1 | 1 | 1 | 0 | 10 |
| Du | 1 | 1 | 0 | 1 | 1 | 1 | 2 | 1 | 1 | 1 | 1 | 11 |
| Sheng | 1 | 0 | 1 | 1 | 1 | 1 | 2 | 1 | 1 | 0 | 0 | 9 |
| Xie | 0 | 1 | 0 | 1 | 1 | 1 | 2 | 1 | 0 | 1 | 1 | 9 |
| Baydursahin | 0 | 1 | 0 | 1 | 1 | 1 | 0 | 1 | 1 | 1 | 0 | 7 |
